# CHARACTERIZING CARE PARTNER INVOLVEMENT IN THE COMMUNITY AGING IN PLACE, ADVANCING BETTER LIVING FOR ELDERS PROGRAM

**DOI:** 10.1093/geroni/igae098.0762

**Published:** 2024-12-31

**Authors:** Kate Perepezko, Pam Toto, Beth Fields

**Affiliations:** Scripps Gerontology Center, Lansdale, Pennsylvania, United States; University of Pittsburgh, Pittsburgh, Pennsylvania, United States; University of Wisconsin Madison, Madison, Wisconsin, United States

## Abstract

A growing number of interventions exist to help older adults age safely and independently in their homes, however, many of these programs overlook care partners (i.e., family or friends who provide support). The purpose of the current study was to report on the types of care partner involvement in the Community Aging in Place-Advancing Better Living for Elders (CAPABLE) program. We characterize care partner involvement using multiple data sources: (1) Number of interventionist contacts, (2) Number of goals set, and (3) Quality of care partner interactions with the older adult. We used personas to represent the major types of care partner involvement observed (Miaskiewicz & Kozar, 2011). Preliminary analysis revealed three types of care partners, “Passive Peter,” “Collaborative Candice” and “Safety-Conscious Savannah.” Passive Peter had minimal communication throughout the program (n=1) and did not identify any goals. Collaborative Candice had some contact with interventionists (n=3) but couldn’t attend all visits because she worked full time. Interventionists described her as “working well” with the older adult. She identified two goals for herself. Finally, Safety-Conscious Savannah was involved in one intervention visit and spoke with interventionists on the phone (n=2). She provided support for the older adult’s goals and identified her own goal that was focused on the older adult’s safety. Interventionists described her as doing a lot of work for the older adult. These findings reveal different ways that care partners are involved in an aging in place intervention and can inform future interventions aiming to include care partners.

